# Rapid Genome Mapping in Nanochannel Arrays for Highly Complete and Accurate *De Novo* Sequence Assembly of the Complex *Aegilops tauschii* Genome

**DOI:** 10.1371/journal.pone.0055864

**Published:** 2013-02-06

**Authors:** Alex R. Hastie, Lingli Dong, Alexis Smith, Jeff Finklestein, Ernest T. Lam, Naxin Huo, Han Cao, Pui-Yan Kwok, Karin R. Deal, Jan Dvorak, Ming-Cheng Luo, Yong Gu, Ming Xiao

**Affiliations:** 1 BioNano Genomics, San Diego, California, United States of America; 2 Genomics and Gene Discovery Research Unit, United States Department of Agriculture - Agricultural Research Service, Albany, California, United States of America; 3 Department of Plant Sciences, University of California Davis, Davis, California, United States of America; 4 Institute for Human Genetics, University of California San Francisco, San Francisco, California, United States of America; Kansas State University, United States of America

## Abstract

Next-generation sequencing (NGS) technologies have enabled high-throughput and low-cost generation of sequence data; however, *de novo* genome assembly remains a great challenge, particularly for large genomes. NGS short reads are often insufficient to create large contigs that span repeat sequences and to facilitate unambiguous assembly. Plant genomes are notorious for containing high quantities of repetitive elements, which combined with huge genome sizes, makes accurate assembly of these large and complex genomes intractable thus far. Using two-color genome mapping of tiling bacterial artificial chromosomes (BAC) clones on nanochannel arrays, we completed high-confidence assembly of a 2.1-Mb, highly repetitive region in the large and complex genome of *Aegilops tauschii*, the D-genome donor of hexaploid wheat (*Triticum aestivum*). Genome mapping is based on direct visualization of sequence motifs on single DNA molecules hundreds of kilobases in length. With the genome map as a scaffold, we anchored unplaced sequence contigs, validated the initial draft assembly, and resolved instances of misassembly, some involving contigs <2 kb long, to dramatically improve the assembly from 75% to 95% complete.

## Introduction

Accurate *de novo* assembly of sequence reads represents the weak link in genome projects despite advances in high-throughput sequencing [1], [2] . There are two general steps in genome sequence assembly: generation of sequence contigs and scaffolds, and their anchoring on genome-wide, lower resolution maps. NGS platforms generate sequence reads ranging from 25 to more than 500 bases [3], while reads of up to 1000 bases can be obtained by Sanger sequencing with high accuracy. NGS reads are often too short for unambiguous assembly. Paired-end reads can bridge contigs into scaffolds, but there are often gaps within the scaffolds. To order contigs and scaffolds, high-resolution genomic maps from an independent technology platform are needed. They may be of chromosomal scale, i.e., genetic maps, or regional scale, i.e., contigs of bacterial artificial chromosomes (BACs) or fosmids [4]. Contigs and scaffolds may be difficult to map if they are too short compared to the map resolution. For example, maps may have a resolution of 50–150 kb while many contigs and scaffolds may only span a few kilobases. Additionally, there are errors in the contigs and scaffolds themselves, often due to misassembly of repeat sequences. Typical medium to large genomes contain 40–85% repetitive sequences [5]–[8], dramatically hindering effective *de novo* sequence assembly.

Genome finishing has relied on guidance of a physical map for large and complex genomes, including human, arabidopsis [9], rice [10] and maize [11], [12]. BAC-based restriction fragment physical mapping of complex genomes is fairly robust because even in the presence of interspersed repeat sequences along the BAC inserts (typically 100–220 kb long) a unique pattern of restriction fragments is generated. The state of the art technologies for physical map construction include SNaPshot 13,14, whole-genome profiling [15], [16], optical mapping [17], [18] and genome mapping [19]. SNaPshot is a restriction fingerprinting method which uses one or more restriction enzymes and fluorescent labels followed by separation of fragments by capillary electrophoresis. SNaPshot has been used for physical mapping of wheat and other genomes [14], [20]. Optical mapping provides an additional layer of information by retaining the physical order of restriction sites along DNA molecules immobilized on a surface [18]. It has been applied to the maize and the rice genome [11], [21]. One can validate a sequence assembly by comparing *in silico* sequence motif maps to consensus optical maps [22]–[25]. However, information density for optical maps is only about one site per 20 kb, and the technology is limited in utility by high error-rates, non-uniform DNA linearization, and low throughput. Therefore, a high-resolution (<5 kb), DNA sequencing-independent mapping method that can overcome these constraints of optical mapping is much needed.

Genome mapping on nanochannel arrays at the single-molecule level overcomes many of the limitations of preexisting technologies and has recently been described in depth [19]. This technology uses nicking enzymes to create sequence-specific nicks that are subsequently labeled by a fluorescent nucleotide analog [26]. The nick-labeled DNA is stained with the intercalating dye YOYO-1, loaded onto the nanofluidic chip by an electric field, and imaged with a CCD camera. The DNA is linearized by confinement in a nanochannel array [27], resulting in uniform linearization and allowing precise and accurate measurement of the distance between nick-labels on DNA molecules comprising a signature pattern. Also, the DNA loading and imaging cycle can be repeated many times in a completely automated fashion; data can be obtained at a high throughput of ∼5 Gb/hour. Genome mapping was previously used to map the 4.7-Mb, highly variable human MHC region. It was able to distinguish haplotype differences, identify a segmental duplication, and identify errors in the reference assembly [19].

The tribe Triticeae includes wheat and the closely related genus Aegilops, the source of two of the three wheat genomes. Diploid Triticeae genomes range from <4 to >8 Gb, and approximately 90% of their genomes is comprised of repetitive sequences [28]. For example, *Ae. tauschii* contains 91% repetitive elements, 2.5% known genes and 6% low-copy sequence [29]. Shotgun genome sequencing of several Triticeae species has been attempted, but the resulting assemblies remain incomplete and coverage is uneven, limiting analysis to mostly gene-rich regions [30]–[33]. The current consensus is that these genomes can only be tackled with an ordered clone sequencing approach. This approach is being adopted by the International Wheat Genome Sequencing Consortium (IWGSC, www.wheatgenome.org) and other genome sequencing projects involving species in this tribe. For ordering and selecting BAC clones, a physical map is constructed with SNaPshot fingerprinting [13], [14] or whole-genome profiling [15], [16]. A set of minimal tiling path (MTP) BAC clones is selected to maximize coverage while controlling for redundancy. The MTP BAC clones are sequenced as pools, and the sequences are scaffolded with the ultimate goal of creating a complete and high-fidelity *de novo* genome assembly.

We have further extended genome mapping for use with a second nicking enzyme and a second label color. This new strategy greatly improves information density of genome mapping. We have applied it to a 2.1-Mb prolamin gene family region from the genome of *Ae. tauschii*, the D genome donor of hexaploid bread wheat. This region is rich in syntelogs, and its assembly is exceptionally challenging. With the improved genome mapping technology, we have constructed a high-resolution physical map and used it to correct the physical map of the region and used it to validate and correct the physical map generated by SNaPshot fingerprinting technology. We then used the genome map to facilitate *de novo* sequence assembly of the region by anchoring sequence scaffolds, validating correctly assembled regions and correcting inaccuracies in the scaffolds, and producing a highly accurate and complete sequence assembly.

## Results

### Generation of a two-color genome map with two nicking enzymes

**Figure 1:**
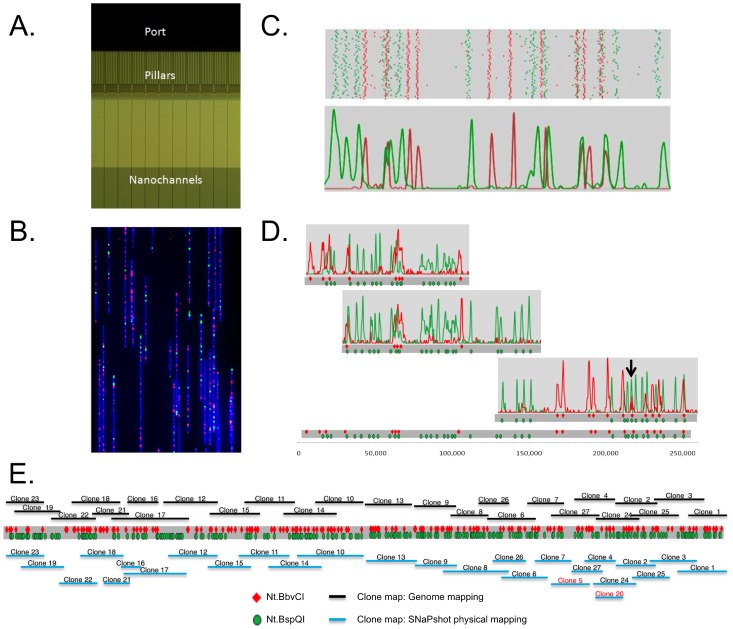
Two-color genome mapping with two enzymes. A. The DNA backbone is stained with YOYO-1 and loaded into the port of a nanochannel array chip. The DNA molecules are introduced into the region with pillars and micron-scale relaxation channels by an electric field where they unwind and linearize. Finally, they are moved into the 45 nm nanochannels, where they stretch uniformly to 85% of the length of perfectly linear B-DNA. B. Linearized BAC DNA molecules in nanochannels. The DNA molecule is stained with YOYO-1, and Nt.BspQI and Nt.BbvCI nicks are labeled with green and red dyes, respectively. C. Molecule length and nick locations are extracted from the images by custom image-analysis software. By clustering individual molecules with high similarity of green label patterns, distinct patterns are extracted (top panel). The locations of the red labels are then overlaid on the green label patterns (middle pattern). A histogram plot of the above clusters is shown in the bottom panel. The peaks represent the location of each sequence motif (GCTCTTC and CCTCAGC) along the linearized DNA molecules. D. Consensus maps for individual BAC clones are shown. Consensus maps are combined by using overlapping patterns, and the final genome map is shown at the bottom. E. The clone map from genome mapping is shown at the top and the full genome map as a grey bar with Nt.BspQI and Nt.BbvCI motif locations in green and red. Below the genome map, in blue, is the physical map from SNaPshot fingerprinting. The total length of the genome map is 2.1 Mb.

We constructed a genome map using two nicking enzymes, Nt.BbvCI and Nt.BspQI, whose nick motifs were labeled with red and green dyes, respectively, across 27 BACs making up an MTP of a 2.1-Mb region containing the prolamin multigene family in the *Ae. tauschii* genome. Figure 1A shows the layout of the IrysChip. The YOYO-stained DNA was loaded into the port, unwound within the pillar structures, and linearized inside the 45 nm nanochannels (Figure 1B). After image processing, individual BAC molecules with red and green labels distributed at sequence-specific locations were compared and clustered into a pools with similar map patterns (Figure 1C, top). Density plots for the BAC clones were generated to determine the consensus peak locations (Figure 1C, bottom). These consensus maps of individual BAC clones were aligned based on overlaps of consensus maps of adjacent BACs (Figure 1D) to create a genome map of the entire region. The two-color labeling strategy resulted in an average information density of one label per 4.8 kb (437 labels in 2.1 Mb). Since each motif was marked by its own color, peaks of different motifs could be distinguished from each other even if their peaks were almost overlapping (arrow in Figure 1D). Peaks of the same motif (the same color) could be resolved when they were at least ∼1.5 kb apart, as previously established [19]. Taking advantage of the combination of long molecule lengths (∼140 kb average), high-resolution, accurate length measurement, and multiple sequence motifs, we generated a high-quality genome map of the 2.1-Mb region for scaffold assembly.

### Validation of SNaPshot-based physical map by genome mapping

The entire genome map for the 2.1-Mb region is shown in Figure 1E. The predicted positions of BAC clones based on the genome map are above the map. There were no overlapping nick motifs between clones 10 and 13 and between clones 13 and 9. However, sequence overlap between the three clones confirmed tiling and allowed for assembly of the region. BAC clone positions in the 2.1-Mb region were also determined by SNaPshot fingerprinting and shown for comparison (bottom of Figure 1E). The genome map-based and the SNaPshot-base clone positions and overlaps were concordant for most clones. SNaPshot does not provide precise clone sizes or overlap lengths because it generates an incomplete collection of short and unordered fragments for a BAC. Therefore, the clone positions and boundaries are much more accurate on the genome map than on a physical map generated by SNaPshot fingerprinting.

Of the 27 BAC clones analyzed by genome mapping, 25 had the same placement as in the SNaPshot map. Genome mapping suggested that clones 5 and 20 did not belong in this 2.1-Mb region. Clone 5 was reanalyzed by SNaPshot and found to differ from the clone originally used for MTP construction, implicating contamination during clone picking for sequencing. SNaPshot fingerprinting of clone 20 was consistent with the original SNaPshot result. However, upon reevaluation, the clone was found to have a weak score for placement on the SNaPshot physical map and, in agreement with the genome map result, was most likely misplaced during BAC contig assembly.

### Genome map-based assessment of *de novo* sequence assembly

**Figure 2:**
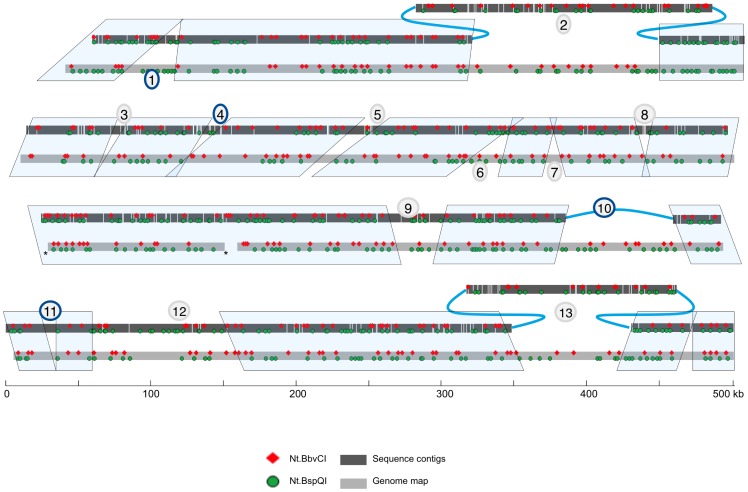
Comparison of the sequence assembly scaffold to the genome map. The sequence assembly is shown with dark grey boxes representing sequence contigs. Contigs were bridged by paired-end sequence reads. The genome map is represented by light grey boxes. Shaded boxes around regions of both maps denote regions where the sequence assembly matches the genome map well. Regions where there are significant discrepancies are numbered and discussed in the results section. The two gaps in the genome map are denoted with asterisks.

The minimal tiling path for this region contained 23 BAC clones, and they were initially sequenced using the 454 platform. For constructing a contiguous physical map using genome mapping, four additional clones were also analyzed but were not sequenced. The sequence reads were assembled and scaffolded with 3-kb paired-end reads. Several clones (5, 7, 9, 14 and 20) were not included in the paired-end sequencing because they were selected later for additional coverage. BAC end-sequences and genetic markers were also used to improve scaffolding, and this resulted in sequence scaffolds that were ordered along the genetic map, covering 2.1 Mb. A total of 254 contigs were joined primarily by paired-end reads into a scaffold. Several small scaffolds could not be placed within the main scaffold. Figure 2 shows the *in silico* map for Nt.BspQI and Nt.BbvCI motifs in the main scaffold. Regions numbered 1 to 13 (Figure 2) showed discrepancies. About 75% of the genome map could be aligned with sequence scaffolds (region 12 was not included in this calculation; see Results section “Genome map guided *de novo* sequence assembly” for explanation).

### Genome map guided sequence assembly: removing incorrectly scaffolded sequence contigs in *de novo* assemblies

**Figure 3:**
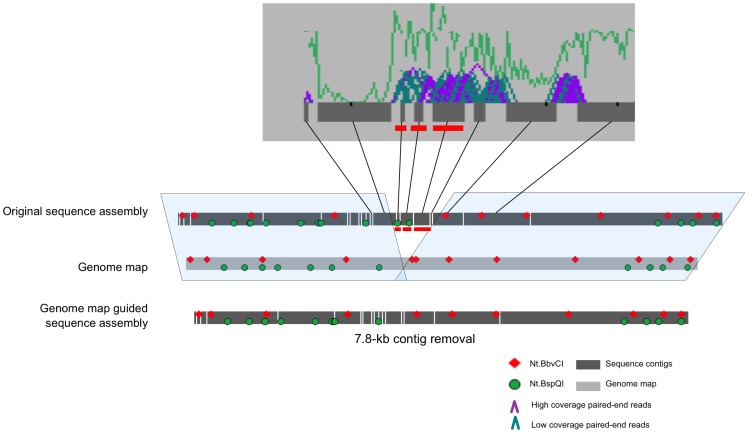
Deletion of incorrect contigs in genome map-guided *de novo* sequence assembly. The original assembly contained two Nt.BspQI sites and ∼8 kb of sequence that were absent from the genome map. The top image is output from gsAssembler and shows the scaffolding of contigs using paired-end reads. The green line represents the sequence coverage for each region. Paired-end reads are represented by pink (high coverage) and aqua (low coverage) carrots (&squ;). The three contigs with red bars beneath them contain the extra sequence motifs and total sequence consistent with the predicted incorrect scaffold. They also contain weak paired-end data indicating that the contigs are misplaced. The bottom line shows the sequence assembly after deletion of the three contigs with red bars.

The most common and easily identified type of discrepancy between the genome map and the scaffold came from regions where sequence contigs were incorrectly inserted into the scaffold. In these cases, motif sites were present in the scaffold but absent in the genome map while the flanking regions matched well (as in Figure 2, #2, 4, 5, 8 and 9). This type of discrepancy can also be identified based on length measurements alone, as in Figure 2, #3. Figure 3 shows the scaffolding results generated with gsAssembler using the paired-end reads. Two of the three contigs with red bars beneath them contained Nt.BspQI sites that were absent in the genome map and the length of the three together is equal to the total discrepancy between the genome map and the assembly. Additionally, the contigs had weak paired-end coverage; therefore, they were likely incorrectly scaffolded and were removed from the assembly. The resulting scaffold mapped correctly to the consensus genome map.

### Genome map guided sequence assembly: filling gaps in *de novo* assemblies

**Figure 4:**
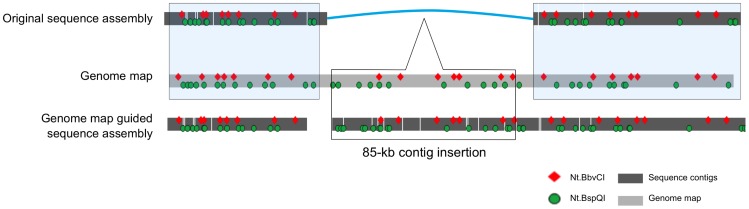
Insertion of unassembled contigs into gaps in genome map-guided *de novo* sequence assembly. The top line shows the *in silico* map for the original sequence assembly; the genome map is shown in the middle. An 85-kb segment of DNA was missing from the sequence assembly. The corresponding DNA sequence was added to the assembly by anchoring a previously unplaced scaffold on the genome map. The resulting corrected assembly is shown in the bottom row.

A number of the inconsistencies between the genome map and the scaffold were due to gaps in the sequence scaffold (Figure 2, #1, 6, 7, 10). The largest gap was 85 kb (Figure 2, #10; Figure 4), which belonged to a region with incomplete paired-end information (BACs 7 and 9 did not have paired-end reads). In order to fill this gap, we used the genome map directly as a scaffold. We searched for the missing sequence in unplaced sequence contigs and scaffolds from the original sequence assembly. An 85-kb scaffold that did not contain a BAC end sequence matched the genome map in the gap; we placed this scaffold in the gap denoted with a triangle in the original scaffold (Figure 4). The final assembly is shown in the bottom line with the genome map guided insertion marked with a box in the middle. The right junction of the insertion was confirmed by PCR (data not shown).

### Genome map guided sequence assembly: identifying misassembled contigs in *de novo* assemblies

**Figure 5:**
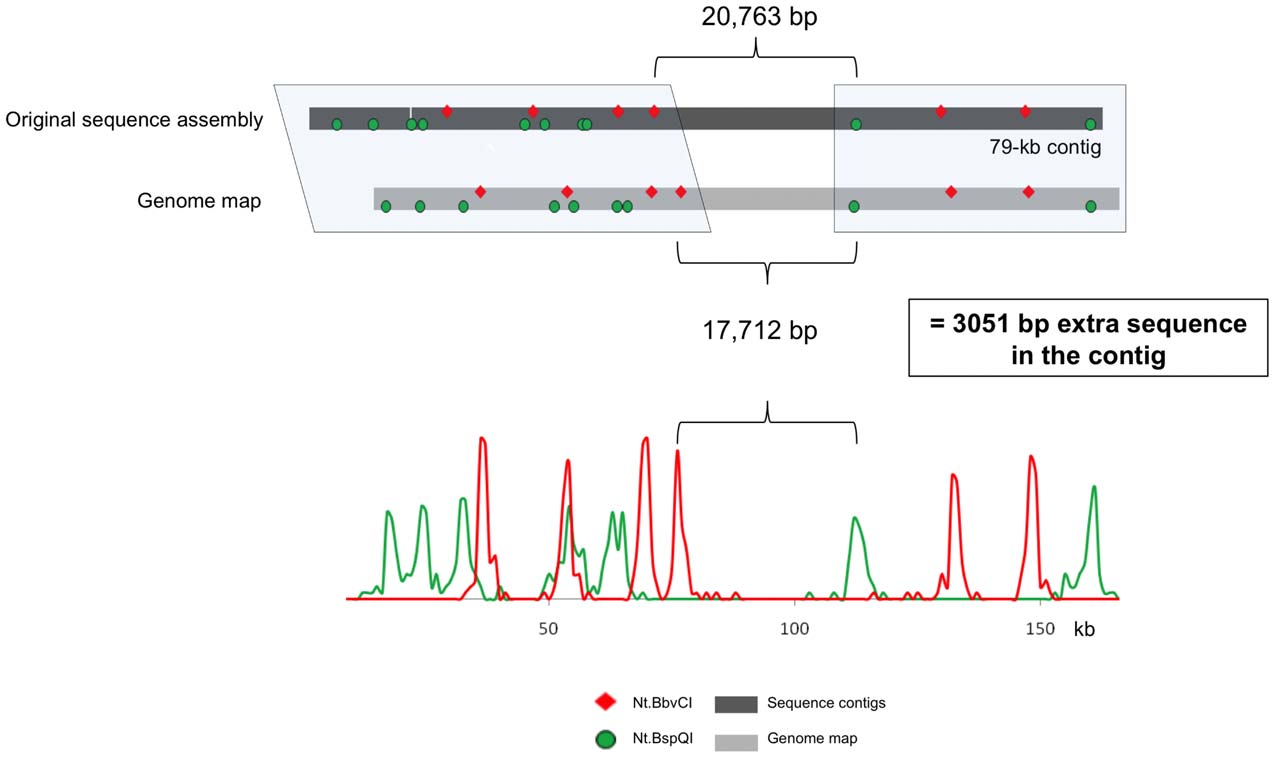
Contig assembly error identification through genome map comparison. The top line represents the *in silico* map for the original sequence assembly, the majority of which is covered by a single sequence contig. The genome map matches on the left and right sides of the contig (shown with shaded boxes). ∼3 kb of sequence was incorrectly inserted into the contig during assembly.

In addition to scaffolding errors, we observed errors in the sequence contig assembly (Figure 2, #2 and 11). Figure 5 represents a zoomed-in view of example 11 from Figure 1. The first ∼40 kb and the last ∼30 kb of this assembled region matched the genome map (boxed regions in Figure 5). This region was covered by a single, 79-kb sequence contig. Based on the contig sequence, the distance between the marked Nt.BbvCI site and the adjacent Nt.BspQI site was 20,763 bp. However, it was measured to be 17.7 kb in the genome map. The histogram for the consensus genome map is shown. It has robust peaks giving high confidence in the distance measurement. This region is primarily made up of LTR elements and is likely prone to assembly errors.

### Genome map guided sequence assembly: identification and reassembly of satellite repeated sequences

**Figure 6:**
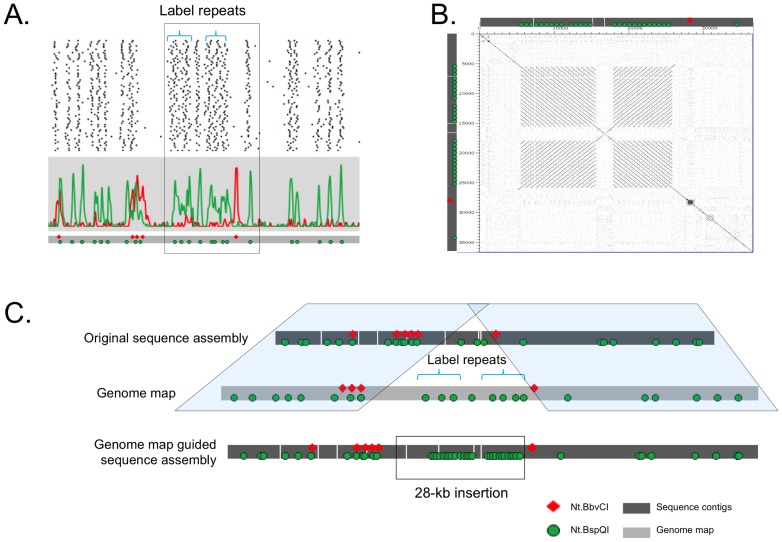
Identification and assembly of repeat sequences in genome map-guided *de novo* sequence assembly. Panel A shows the strip diagram for one of the clones that covers the high density region, each line represents a different molecule and the spots are the location of green (Nt.BspQI) labels. Two high-density label regions are marked “label repeats,” and they do not cluster into discrete peaks. Panel B shows a pairwise alignment of the high-density region after reassembly based on the genome map. The alignment shows two blocks of direct repeats which are inverted with respect to one another. Panel C shows the original assembly on the top, the genome map in the middle and the final assembly on the bottom, containing the repeat sequence as predicted by the genome map.

At position 1 in Figure 2, there was a 28-kb gap in the scaffold. The consensus genome map showed two blocks of DNA, each containing a very high density of Nt.BspQI sites beyond our optical resolution. Figure 6A shows the strip diagram for one of the clones that covers the region; each line represents a different molecule and the green spots are locations of the Nt.BspQI motif. The region marked “label repeats” (Figure 6) did not cluster into discrete peaks because not all close labels were detected as separate. This high-density nick motif commonly represents a tandem sequence repeat (satellite) and in this case, we found two blocks of potential tandem repeats. In order to appropriately assemble the sequence, we found the repeat sequence in an unplaced contig. The repeat was 670 bp long with 95–99% identity and contained an Nt.BspQI site. The original assembly had only a single occurrence of the 670-bp sequence cassette. By extracting all of the sequence reads that contained the cassette, incorporating additional reads generated by Sanger sequencing and separately reassembling the region using Consed, we were able to assemble two regions containing direct repeats of lengths 9.8 kb and 8 kb, in good agreement with the measured lengths of the high density Nt.BspQI regions. Figure 6B shows a pairwise alignment of the repeat structure with two blocks of direct tandem repeats which are inversely oriented with respect to each other. The genome map-independent sequence assembly, where it is consistent with the consensus genome map, is shown by shaded boxes in Figure 6C. The label repeats are noted in the genome map and absent in the scaffold. After insertion of 28 kb of reassembled contigs, the assembly was in good agreement with the genome map.

### Genome map guided *de novo* sequence assembly

**Figure 7:**
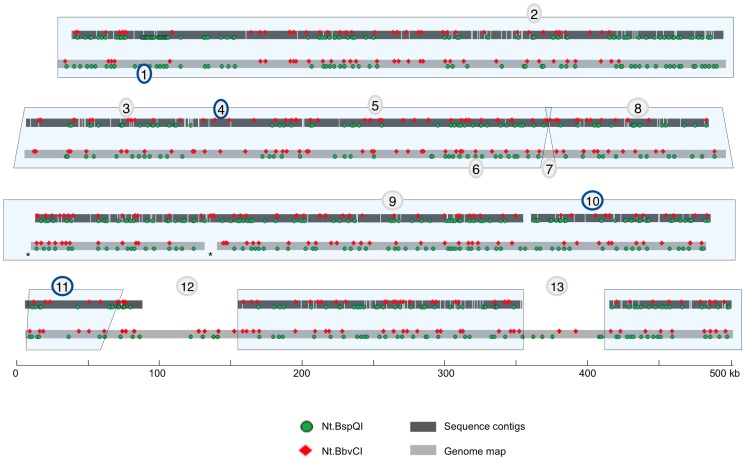
Comparison of the final genome map guided sequence assembly to the genome map. The final sequence assembly almost completely spans the genome map. Gaps in the genome map are denoted with asterisks.

After correcting the sequence scaffold (Figure 7, #1–6, 8–13), as discussed in the examples above, and excluding the gap at position 12 (which resulted from missing sequenced clone coverage), we have generated a high-confidence assembly that covered 95% of the consensus genome map. We were unable to fill the gap at position 7 because the region contained no sequence motifs; however, we measured this gap to be 12.79 kb. Position 12 is in the region that clone 5 should have filled, based on SNaPshot (Figure 1). The genome mapped clone 5 was deemed contaminated, but it was unclear if the correct or contaminated BAC was sequenced. Much of the sequence at this region (position 12) matched the clone 5 genome map, suggesting that the contaminated clone 5 was also sequenced and then misassembled into the scaffold. Since clone 27 actually overlaps with clones 4 and 7 but was not sequenced, properly filling this gap is not possible without additional sequencing. Discrepancy 13 was partially corrected but remains incomplete. Filling the remaining gaps will require sequencing of additional BACs that were not originally part of the MTP in this region.

## Discussion

Having a high-quality reference genome assembly for an organism is critical to the understanding of its biology and evolutionary relationship with other organisms. With a good reference sequence assembly, resequencing with NGS can be inexpensive and useful for studies of genetic variation. *De novo* assembly of additional genomes may allow more comprehensive surveys of variation, especially structural variation, as it relieves some of the biases associated with reference-dependent alignment and variant calling [34]. However, whole genome assembly is often difficult and cost-prohibitive. To date, very few high-quality assemblies are available for large and complex genomes [2], and additional *de novo* assemblies beyond a single reference assembly are uncommon. Generated using BioNano Genomics’ nanochannel array mapping technology, genome maps can guide sequence assembly. It fills a void in *de novo* assembly strategies by economically providing a high-resolution physical map that can be used for anchoring contigs and scaffolds (Figure 4) and capable of identifying misassembled contigs (Figures 3, 5 and 6), thus dramatically improving the fidelity of the final assembly (Figure 7).

Genome mapping has several advantages over SNaPshot fingerprinting for physical map construction. With SNaPshot fingerprinting, the sized fragments used for the physical map construction are only a portion of all fragments generated from a simultaneous, 5-enzyme digestion. Small (<70 bp), large (>1000 bp), those with non-labeling ends (HaeIII – HaeIII cleavage) and those from high-copy repeats are not sized and therefore not included in the fingerprint. An additional impediment is that the order of the fragment along a molecule is not known. In genome mapping, clone sizes are directly measured using the YOYO-stained DNA backbone. Since the DNA molecules are not cut but labeled at nick sites, it provides relative locations and linear order of sequence motifs on the DNA molecule. As a result, genome mapping produces accurate size estimates and facilitates tiling as shown in our ability to improve (and in some instances correct) the SNaPshot physical map. Additionally, multiple BACs can be analyzed in pools to increase throughput. Whole-genome profiling is a sequence tag based approach that is high throughput but since it is based on sets of sequence tags adjacent to restriction sites, it is unable to provide any length measurements. Optical mapping of static substrate-affixed DNA molecules has been used to construct physical maps for large and complex organisms [18], [21]. It suffers from low throughput, high error-rates and inconsistent DNA stretching. It is also a highly specialized technique that is difficult to master and therefore used by few labs. Genome mapping uses a nanochannel array to reproducibly and uniformly linearize DNA. In addition to the improved noise characteristics, by virtue of keeping DNA in solution rather than affixed, the system can perform cycles of channel-loading and imaging to generate throughputs of at least 5 Gb of DNA per hour. As shown here, genome mapping has the fundamental advantage that multiple motifs can be labeled with different colors, significantly increasing the information density.

An important advantage of BAC-by-BAC sequencing strategies is that a physical map, with BAC positional information, is available prior to the start of genome sequencing. Physical maps provide very long and rigid scaffolds with BAC resolution and allow sequence contigs and paired-end scaffolds, from a limited number of pooled BACs, to be placed in a defined region. Distance information from physical map restriction fragments and BAC overlap have been used to guide sequence assembly by incorporating length constraints on the contig assembly [35], [36]. This improves assembly, but these restriction maps are unordered and thus provide limited information. None of these impediments are present in genome mapping, which can therefore be used to guide and validate *de novo* assembly of sequencing data.

DNA sequence contigs from shotgun sequencing for *de novo* assembly are usually small due to the presence of repetitive elements (such as satellites, tandem repeats and retroelements) and other sequence duplications. Assembly of contigs into scaffolds can be aided by the use of paired-end reads. However, we have shown even with a physical map generated by SNaPshot, relatively long sequence reads and short paired-end reads, the sequence assembly was only about 75% complete. By incorporating genome mapping, we improved the assembly to about 95% complete. The improvement stemmed from a number of factors: genome mapping is DNA sequencing-independent, it is high resolution (1.5 kb), has accurate length measurements (within 1 kb [19]) and high information density (one site/5 kb). Genome mapping is ideally suited for validating, improving and facilitating anchoring of sequence contigs and paired-end scaffolds for full genome assembly.

One of the most difficult challenges in sequence assembly is caused by highly repetitive sequences. Repetitive sequences often collapse in assembly due to the high sequence identity. Therefore, it is often impossible to assemble them correctly with sequence information alone. Genome maps can span long repeat regions not containing nick motifs. Even if sequence assemblies cannot be anchored directly to the repeat region, the repeat unit identity can be identified by use of adjacent mapped sequence contigs and the length of the repeat containing region can be measured on the genome map. By doing so, unambiguous contigs on each side of the repeat stretch can be linked with the aid of a genome map. Additionally, some repeat sequences contain nick motif sites and can be predicted by a characteristic genome map pattern such as a high density of labels, as in Figure 6 or a repetitive pattern such as may be seen in a segmental duplication. This information can guide more accurate assembly of difficult and complex regions such as tandem gene duplication regions that are present in eukaryotic genomes.

In summary, we envision adoption of genome mapping in whole genome *de novo* sequence assembly projects as it fills two critical needs, providing and/or correcting the physical map-based minimal tiling path and guiding *de novo* sequence assembly. Genome mapping can be used for *ab initio* physical map generation from BACs or from genomic DNA. BAC pools or genomic DNA can be sequenced with NGS, and assemblies can be guided with the genome map. This process would greatly improve the fidelity of the assembly process by detecting and correcting incorrect assemblies at an early stage. The genome map can also be used to recognize certain structural elements such as tandem repeats, which cause problems during assembly, and guide their assembly. Based on our current throughput of ∼5 Gb per hour, we expect to be able to collect data for 20x coverage of the *Ae. tauschii* genome in less than one day. BACs can be accomplished in a greatly reduced time frame than with the current methods. We expect this workflow to provide an unprecedented level of completion and accuracy in *de novo* genome sequence assembly.

## Materials and Methods

### Sample preparation and data collection

A total of 663,000 BAC clones were fingerprinted with the SNaPshot high-information-content-fingerprinting (HICF) technology [13]. Minimum tiling path BACs (27 total) were selected to cover a ∼2-Mb region; clones 1–27 are: RI628H11, RI339A11, HD254F14, HD330o06, MI263M23, HI297K16, MI236G21, RI313E10, TCM018D08, HI219K02, HD251I24, RI339P08, RI374K13, MI305M04, RI591E12, HD057J22, RI346M11, RI575G17, HD525B13, N_BB038E03, HI050E23, HD470O12, RI543P09, HI242N01, RI524D20, RI549B21, HD451L17, HD321F15.

All DNA samples used in the study were prepared using the Qiagen Large-Construct Kit. To prepare BAC mixtures, we grew 250 mL cultures of each BAC in LB containing 20 µg/mL chloramphenicol or 12.5 µg/mL tetracycline overnight and combined the separate cultures before proceeding with DNA extraction of the BACs as a pool. The DNA samples were quantified using Quant-iTdsDNA Assay Kit (Invitrogen/Molecular Probes) and their quality assessed using pulsed-field gel electrophoresis. One microgram of BAC DNA was nicked with the nicking endonuclease, Nt.BspQI (New England BioLabs, NEB). Nicked DNA was labeled with Alexa546-dUTP (Invitrogen) and Taq polymerase (NEB). After labeling, the nick was ligated by adding dNTPs and T4 ligase (NEB). DNA was purified. The second nick labeling step was performed the same as the first except Nt.BbvCI was used and the nick was labeled with Alexa647-dUTP. DNA was linearized with Cre recombinase and LoxP containing dsDNA oligonucleotides. Cre was removed with Qiagen protease (Qiagen). The backbone of fluorescently tagged DNA was stained with YOYO-1 (Invitrogen).

DNA was loaded in BioNano Genomics nanochannel array chips by electrophoresis of DNA, automated by the Irys system. Twelve volts were applied to concentrate the DNA, 30 V were applied to move DNA into the nanochannels, and 10 V was applied to distribute the DNA in the nanochannels. Linearized DNA molecules were imaged using blue, green and red lasers for YOYO-1, Alexa546, and Alexa647 on the BioNano Genomics Irys system.

### Genome map analysis

The entire DNA molecule (YOYO-1) and locations of fluorescent labels (Alexa546 and Alexa647) along each molecule were detected using the in house software package, IrysView. A set of label locations of each DNA molecule comprises the individual DNA molecular map.

Single-molecule Nt.BspQI maps were clustered, as previously described [19]. Briefly, all single-molecule maps were scored for similarity to one another in a pairwise comparison, and a Euclidian distance matrix was built. Maps were then clustered with the R package, *fastcluster*. From the clusters, the label locations were plotted as histograms and the peaks identified by fitting a Gaussian curve and used to make consensus maps. The Nt.BbvCI label positions were overlaid on the Nt.BspQI clusters, histograms were plotted, and peaks were fitted to produce a two-color map.

### Next-generation sequencing of BAC clones

BAC clones 1–23 were sequenced using the Roche/454 Titanium platform (using GS FLX or GS FLX+ chemistry) in barcoded pools with an average of five BACs per pool. The average read length was 570 bases with 20x coverage depth. gsAssembler was used for de novo assembly; after testing several stringency parameters to optimize assembly, we used reads with 40-bp overlap and a 95% identity threshold. For the GS FLX method, the average contig size is 5116 bp, N50 is 12937 bp and the largest contig size is 47797 bp. For the GS FLX+ method, the average contig size is 15758 bp, N50 is 40861 bp, and the largest contig size is 93822 bp. Eighteen of the BACs (1–4, 6, 8, 10–13, 15–19, 21–23) were pooled together with an additional ∼300 non-contiguous BACs to make one paired-end library with an average insert size of 3 kb and then sequenced by Roche/454 for 10x average coverage per BAC. The region of the MTP not covered with paired-end reads is minimal. We used these paired-end reads to scaffold the contigs with a 98% identity threshold, using gsAssembler. A total of 254 contigs and 13 scaffolds were generated. BAC end sequences were used to orient the contigs and scaffolds on the physical map.

### DATA ACCESS

The NCBI accession number for the final sequence (after genome map assisted assembly) is: JX295577.
